# Regional disparity of certified teaching hospitals on physicians’ workload and wages, and popularity among medical students in Japan

**DOI:** 10.1186/s12199-021-00997-3

**Published:** 2021-07-20

**Authors:** Yutaro Ikki, Masaaki Yamada, Michikazu Sekine

**Affiliations:** 1 Center for Postgraduate Clinical Training and Career Development, Nagoya University Hospital, 65 Tsurumai-cho Showa-ku, Nagoya, 466-8560 Japan; 2 Department of Epidemiology and Health Policy, School of Medicine, University of Toyama, 2630 Sugitani, Toyama, 930-0194 Japan

**Keywords:** Workload, Physicians, Regional disparity, Matching program, Japan

## Abstract

**Background:**

Regional disparities in the working conditions of medical doctors have not been fully assessed in Japan. We aimed to clarify these differences in hospital characteristics: doctors’ workload, wages, and popularity among medical students by city population sizes.

**Methods:**

We targeted 423 teaching hospitals certified by the Japanese Society of Internal Medicine and assessed the working conditions of physicians specializing in internal medicine. We calculated their workload (the annual number of discharged patients per physician) and retrieved data on junior residents’ monthly wages from the Resinavi Book which is popular among medical students in Japan to know the teaching hospital’s information and each hospital’s website. Furthermore, we explored the interim matching rate of each hospital as its popularity among medical students. Next, we classified cities in which all hospitals were located into eight groups based on their population size and compared the characteristics of these hospitals using a one-way analysis of variance.

**Results:**

The average workload was 110.3, while the average workload in hospitals located in most populated cities (≥ 2,000,000) was 88.4 (*p* < 0.05). The average monthly wage was 351,199 Japanese yen, while that in most populated cities was 305,635.1 Japanese yen. The average popularity (matching rate) was 101.9%, and the rate in most populated areas was 142.7%, which was significantly higher than in other areas.

**Conclusions:**

Hospitals in most populated areas had significantly lower workloads and wages; however, they were more popular among medical students than those in other areas. This study was the first to quantify the regional disparities in physicians’ working conditions in Japan, and such disparities need to be corrected.

## Background

To address the shortage of medical doctors, the number of medical school graduates has gradually increased since 2008 in Japan [1]. However, the distribution of this surge has been regionally uneven. While the increase in doctors was large in populated cities, it was small in rural areas. Regional disparities in the number of doctors have continued since 2014 [1].

A shortage of doctors caused the existing ones to overwork. In fact, about 40% of doctors working full-time at large-scale general hospitals worked for over 60 h per week [2]. Working overtime could have an impact on their health. Overworked doctors have been reported to have a higher risk of coronary heart disease and stroke [3, 4], mental stress, exhaustion [5], and death from fatigue (*Karoshi*) [6] in Japan. Meanwhile, a heavy workload has a negative impact on patients [7, 8]. Thus, it is a problem of national concern.

Fukuda et al. showed a regional disparity in the number of doctors based on clinical departments [9]. In this research, they analyzed using three regional classifications based on city population: big (≥ 1 million), middle (≥ 200,000), and small city (< 200,000). They found that between 2008 and 2014, the increased rates of physicians specializing in internal medicine in big cities were much higher than those in other regions. To reduce regional disparities, potentially influential factors, such as individual workload, should be assessed.

Since the commencement of the new clinical resident training system in 2004, Japanese medical students can choose hospitals to work (or to receive training). All students are required to be employed at a relatively large-scale, certified teaching hospital by the Ministry of Health, Labor, and Welfare in Japan. Many residents tended and hoped to work in urban areas [10], and the shortage of doctors in rural areas was unresolved. The disparity in the number of doctors might start when medical students enter the workforce. This is because many residents reported to continue working in the same prefecture in their later lives [11]. When these students choose hospitals to work, their characteristics are crucial. A survey showed that they tended to take into consideration the number of clinical cases they might encounter, wages, and the location of hospitals [12]. Therefore, we hypothesized that hospitals in urban areas might provide greater clinical cases and wages with residents than those in less populated ones.

This study aimed to clarify the regional differences in hospital characteristics: doctors’ workloads and wages, and popularity among medical students based on the city population size. Comparing hospital characteristics according to the city size may lead to a key for resolving doctors’ disparity.

## Methods

### Certified teaching hospitals and classification of cities by population size

In this study, we targeted the working conditions of physicians, who specialized in internal medicine. As of 2016, the Japanese Society of Internal Medicine certified 81 and 423 university and large-scale general hospitals as clinical teaching hospitals (“Kyoikubyoin”) respectively [13]. In our research, we analyzed all the latter because university hospitals had significantly fewer clinical cases and lower salaries than general (non-university) hospitals [14]. The large-scale general hospitals are flagship hospitals in each region and distributed nationwide. Subsequently, we classified the cities where all hospitals were located into eight groups based on their populations (< 100,000, < 200,000, < 300,000, < 400,000, < 600,000, < 1,000,000, < 2,000,000, and ≥ 2,000,000). We used data on population from the 2016 government statistics [15]. Overall, 23 wards of Tokyo were defined as the city with the total population.

### Hospital characteristics: beds, physicians’ workload, monthly wages, and popularity

#### Number of beds

The number of beds among hospitals was assessed to identify the hospitals’ scale and their differences.

#### Measure of workload

We referred to an annual report [13] published by the Japanese Society of Internal Medicine in 2017 and retrieved two available indicators: (1) the number of full-time physicians and senior residents specializing in internal medicine and (2) the number of annually discharged patients in internal medicine. We calculated the number of annually discharged patients per physician as a surrogate of physician workload in each hospital. Four hospitals that reported a surreal number of discharged patients were excluded.

$$ \mathrm{Workload}=\frac{\mathrm{annual}\ \mathrm{discharged}\ \mathrm{patients}}{\mathrm{full}-\mathrm{time}\ \mathrm{physicians}+\mathrm{senior}\ \mathrm{residents}} $$

#### Monthly wages of junior residents

We used the data on monthly wages of junior residents (first- and second-year doctors) from a guidance book (Resinavi book) which is popular among medical students in Japan to know the teaching hospital’s information [16] or the website of each hospital. In this study, we included the basic monthly wages and excluded additional pays, such as night shifts, benefits, and bonuses. We omitted 78 hospitals that did not show the basic monthly wages (e.g., only the total wages including night shifts and all benefits); hence, we analyzed 345 hospitals for wages.

#### Measure of popularity among medical students

We used the interim matching rate of hospitals announced from the Japan Residency Matching Program as its hospital popularity among medical students because it demonstrated more accurate numbers of medical students who wished to work rather than the final matching rate. From an interim announcement of the Japan Residency Matching Program [17] in 2016, we retrieved two indicators of each hospital: (1) the number of students who submitted the first choice for a residency program and (2) the fixed (or the maximum) number of the residency program. Next, we used the ratio of the number of first applicants to the fixed number as each hospital’s popularity (the number of first applicants/a fixed number multiplied by 100). If a hospital had some residency programs, such as general and pediatric courses, we utilized the total number of first applicants and the total fixed number of each residency program. Hospitals with higher rates are gaining popularity among medical students. Twenty-six hospitals that did not officially report the interim matching rates were excluded from our analysis. The ratio was treated as a continuous variable.

$$ \mathrm{Popularity}=\frac{\mathrm{first}\ \mathrm{applicants}}{\mathrm{fixed}\ \mathrm{number}}\times 100 $$

### Statistical analysis

We compared the average of hospitals’ characteristics: physicians’ workload, monthly wages, and hospital popularity by eight population groups using one-way analysis of variance, and Tukey’s test was performed to investigate the differences between all groups. The threshold for significance was 2-tailed with *p* values of less than 0.05. All statistical analyses were conducted using the IBM Statistical Package for the Social Sciences version 25.0 (Chicago, IL, USA).

## Results

Table 1 shows the basic characteristics of teaching hospitals. There was no significant difference in the number of beds for inpatients (497.2 on average) and the annual discharged patients (5137.2 on average) at the city level. The average number of physicians and residents was 37.7 and 10.2, respectively. However, significantly more physicians and residents were observed in the hospitals of populated areas (≥ 2,000,000).

**Table 1:** Characteristics of teaching hospitals

City population	Number	Average number of bed (95% CI)	Number of annual discharged patients (internal medicine)	Full-time physicians (internal medicine)	Senior residents (internal medicine)
< 100,000	51	465.9 (422.7–509.2)	4924.3(2855.7–6992.7)	30.9(24.3–37.5)	7.6 (5.3–9.9)
< 200,000	84	474.6 (440.5–508.7)	5407.6 (3358.6–7456.6)	34.1 (29.9–38.3)	8.8 (7.1–10.5)
< 300,000	39	517.0 (469.9–564.0)	5733.8 (4152.2–7315.3)	34.0 (29.8–38.2)	8.2 (5.6–10.9)
< 400,000	40	531.2 (470.9–591.1)	4630.9 (4114.4–5147.3)	35.0 (30.0–40.1)	8.7 (6.1–11.2)
< 600,000	45	514.4 (460.6–568.1)	4914.1 (4159.9–5668.3)	38.4 (31.3–45.6)	8.8 (5.4–12.2)
< 1,000,000	36	493.1 (439.2–546.9)	4917.0 (4151.4–5682.6)	36.7 (30.4–43.1)	8.9 (6.9–10.9)
< 2,000,000	49	481.4 (437.9–524.9)	5292.4 (4540.0–6044.4)	40.8 (35.6–46.0)	11.3 (9.0–13.7)
≥ 2,000,000	79	516.6 (476.3–556.8)	5080.1 (4609.2–5551.1)	47.4^a^ (41.8–53.0)	15.8^b^ (13.0–18.5)
Total	423	497.2 (481.4–513.0)	5137.2 (4621.7–5652.7)	37.7 (35.7–39.8)	10.2 (9.3–11.1)

^a^The number of physicians in ≥2,000,000 was significantly more than that in < 1,000,000 to < 400,000 in Tukey’s test

^b^The number of residents in ≥2,000,000 was significantly more than that in < 1,000,000 to < 1,000,000 in Tukey’s test

**Fig. 1:**
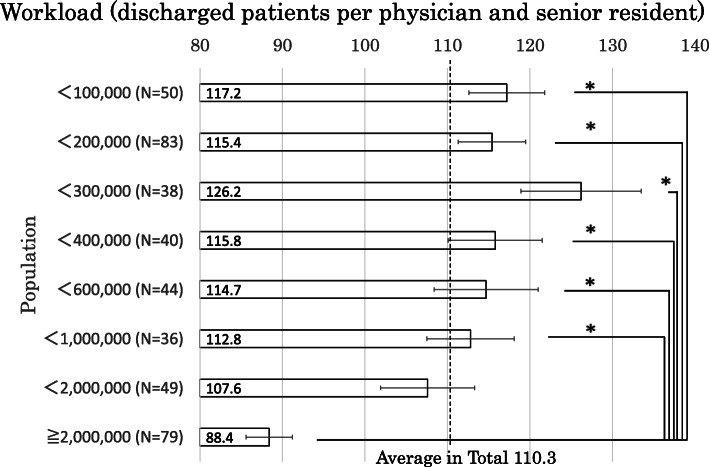
Regional differences in physicians’ workload (the average number of annually discharged patients per physician). The overall average of the 419 general hospitals was 110.3. The hospitals in most populated cities (≥ 2,000,000) had less workload than the average by 21.9 patients (per physician annually) and significantly less workload than in other areas (< 100,000, < 200,000, < 300,000, < 400,000, < 600,000, < 1,000,000). The broken line shows this average number. The box plot shows standard error. *p < 0.05 in Tukey’s test

Figure 1 displays regional differences in physicians’ workload (the average number of annually discharged patients per physician). The overall average of the 419 general hospitals was 110.3. The hospitals in most populated cities (≥ 2,000,000, e.g., 23 wards of Tokyo metropolitan area, Yokohama, Nagoya, and Osaka) had less workload than the average by 21.9 patients (per physician annually) and significantly less workload than in other areas (< 100,000, < 200,000, < 300,000, < 400,000, < 600,000, < 1,000,000).

**Fig. 2:**
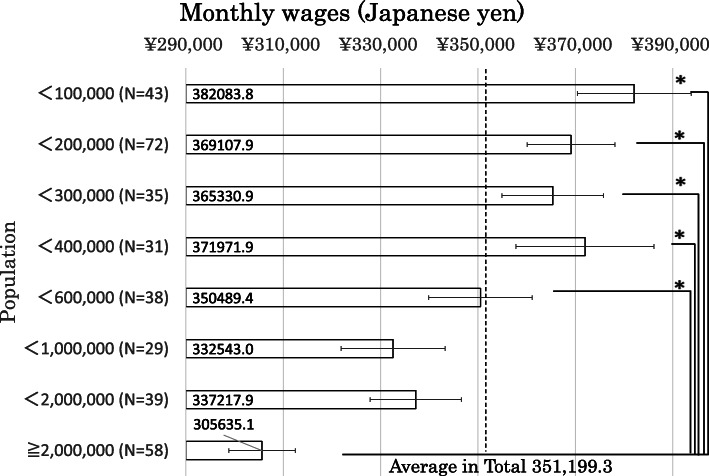
The average monthly wages for each of the eight groups. The average monthly wage among the 345 general hospitals was 351,199.3 Japanese yen. The monthly residents’ wage in the most populated city was 305,635.1 Japanese yen and was significantly lower than in other cities (< 100,000, < 200,000, < 300,000, < 400,000, and < 600,000). The broken line shows this average number. The box plot shows standard error. *p < 0.05 in Tukey’s test

Figure 2 shows the average monthly wages for each of the eight groups. The average monthly wage among the 345 general hospitals was 351,199.3 Japanese yen. The monthly residents’ wage in the most populated city was 305,635.1 Japanese yen and was significantly lower than in other cities (< 100,000, < 200,000, < 300,000, < 400,000, and < 600,000).

**Fig. 3:**
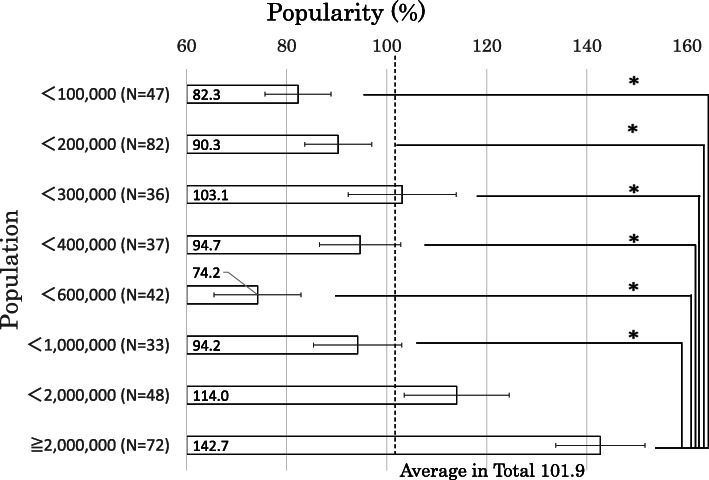
The popularity of medical students according to the interim matching rate. The average ratio among the 397 general hospitals was 101.9%. The ratio in most populated cities (≥ 2,000,000) was 142.7%, which was significantly higher than that in other cities (< 100,000, < 200,000, < 400,000, < 600,000, and < 1,000,000). The broken line shows this average number. The box plot shows standard error. *p < 0.05 in Tukey’s test

Figure 3 presents the popularity of medical students according to the interim matching rate. The average ratio among the 397 general hospitals was 101.9%. The ratio in most populated cities (≥ 2,000,000) was 142.7%, which was significantly higher than that in other cities (< 100,000, < 200,000, < 400,000, <600,000, and < 1.000,000).

## Discussion

We first quantified the regional disparity in hospital characteristics on physicians’ workload, wages of residents, and popularity among medical students by population size in Japan. Contrary to our hypothesis, hospitals in most populated cities had a significantly lower workload per physician (fewer than the total average by 21.9 patients), paid fewer wages (than the average by 43,524.2 Japanese yen), and were more popular among students (than the average by 40.8%). Medical doctors and students were not likely to prioritize the number of cases they might actually experience and not the wages paid. Our findings meant a disadvantage for physicians working in non-populated areas, and that could lead to lower medical services there. Therefore, these regional disparities might be a political issue.

### Workload

In our study, we clarified that physicians working in non-populated areas cared for more patients than those in the populated ones. Physicians working in the city of ≥2,000,000 cared for fewer patients annually by 21.9 patients than the overall average; for fewer by 37.5 patients than physicians working in the city of < 300,000. Although there have been several reports describing the regional disparities in the number of doctors [9, 18] in Japan, the discrepancy in workload has not been explored thus far. To the best of our knowledge, this is the first study to quantify the regional disparity in physicians’ workload in Japan. Excessive workload was reported to be a health hazard [5], burnout [19], and important demotivators for physicians [20, 21]. This problem may cause a public health crisis as well as negative impacts on individual physicians, patients, and healthcare systems [22]. Therefore, physicians working in non-populated areas (particularly de-populated areas) are at a higher risk of developing health problems [9]. It appears difficult to completely resolve these regional workload disparities for each hospital.

The Japanese Medical Specialty Board started a ceiling system [23], restricting the number of senior residents (doctors trained for > 2 years) working in Tokyo, Kanagawa, Aichi, Osaka, and Fukuoka Prefectures from 2018. However, their concentration in these prefectures remained unresolved by the end of 2019; therefore, a stricter ceiling system that can address regional disparity in the number of young doctors as well as the disparity of their workload is needed in Japan.

### Wages

We demonstrated that the average monthly wage of junior residents in the most populated cities was significantly lower than in the others (< 100,000, < 200,000, < 300,000, < 400,000, and < 600,000).

Yamaguchi et al. reported that hospitals in small populated areas in Yamagata prefecture tended to pay doctors more on average than those in the large ones [24]. According to another report in Australia, general practitioners who worked in rural areas had higher earnings [25]. Our results are in line with these findings, showing that residents working in smaller population sizes had a tendency to earn higher wages than those in the larger ones. Wage was ranked as the third important factor in the questionnaire for medical students choosing hospital [12]; however, in fact, residents working in populated cities were paid lower wages than those in other areas. This discrepancy showed that most medical students did not prioritize wages during the resident term, despite hoping for higher salaries.

### Popularity

We also found that there was a significant difference in medical students’ popularity (Fig. 3). The hospitals in the two populated areas were more popular than the average (112.1% in < 2,000,000 and 140.8% in ≥2,000,000). Our results are in line with those of other reports. A survey of medical residents in Japan showed that about 30% of them emphasized the location of hospitals when they decided to choose hospitals for work [12]. Similarly, location was rated as the most important factor for residency selection among American medical students [26]. There were three plausible reasons why medical students wanted to work in urban regions. First, the number of medical students born and raised in populated cities was large; hence, most of them would have returned there. Second, they might think that they could witness a wide range of diseases, including extremely rare ones, through conferences such as clinico-pathological conferences without directly being in charge of these cases at hospitals in populated cities. Finally, the number of famous doctors, surgical operations, and the latest treatments may be accumulated in populated cities. In addition to the ceiling system, others that attract not only young physicians, but also mature ones to work in less populated areas may be needed, such as remote learning, experiencing the latest medicines, or improving working conditions by introducing the Internet of Things.

### Limitations

This study has some limitations. First, we analyzed workload at each hospital; thus, we did not know the actual workload of individual physicians and its disparities within a hospital. We speculated that younger physicians were more in charge of inpatients than older ones. Second, we did not include other factors, such as the existence of famous or teaching doctors and the hospital’s specialty, such as the number of coronary angiography and endoscopy, which may attract medical students. Future studies should include these factors. Third, this study targeted only physicians and doctors specializing in internal medicine. Thus, our findings may not apply to other departments, such as pediatrics or surgery.

## Conclusions

We analyzed hospital characteristics and quantified the magnitude of regional disparities in physicians’ working conditions: workload, residents’ wages, and popularity of teaching hospitals in Japan. Hospitals in most populated cities had significantly lower workloads and paid fewer wages; however, they were more popular among students as compared to the other areas. These regional disparities need to be corrected not only for individual physicians’ health, but also for regional healthcare systems.

## Data Availability

The datasets used and/or analyzed during the current study are included in this published article and available from the corresponding author on reasonable request.

## Abbreviations

ANOVA: Analysis of variance
CPC: Clinico-pathological conference
